# LAUNCHING STANDARDIZED GERIATRIC SURGERY PROGRAMS: THE ALPHA AND BETA PILOTS

**DOI:** 10.1093/geroni/igz038.1488

**Published:** 2019-11-08

**Authors:** Lindsey Zhang

**Affiliations:** American College of Surgeons, Chicago IL, United States

## Abstract

Then Coalition for Quality in Geriatric Surgery has completed both alpha and beta pilots which represent the initial efforts to launch this national quality initiative to improve the care of all older adults undergoing operations. The alpha phase included hospital stakeholders rating the feasibility of implementing standards related to evidence-based high quality surgical care for older adults on the topics of eliciting patient goals, completing a preoperative frailty risk assessment, educating healthcare professional about care specific to older adults, and implementing postoperative age-friendly care models. The beta pilot phase required 9 medical centers nationally (including an academic hospital, an urban medical center, a Kaiser hospital, a VA hospital and a rural hospital) to implement the evidenced based standards for high quality surgical care of older adults. Site verification visits were completed in the Summer 2018 which evaluated the effectiveness of each medical center’s ability to implement each of the standards.

